# Automatic auditory and somatosensory brain responses in relation to cognitive abilities and physical fitness in older adults

**DOI:** 10.1038/s41598-017-14139-9

**Published:** 2017-10-20

**Authors:** Juho M. Strömmer, Nele Põldver, Tomi Waselius, Ville Kirjavainen, Saara Järveläinen, Sanni Björksten, Ina M. Tarkka, Piia Astikainen

**Affiliations:** 1 Department of Psychology, University of Jyvaskyla, Jyväskylä, Finland; 2 Institute of Psychology, Doctoral School of Behavioural, Social and Health Sciences, University of Tartu, Tartu, Estonia; 3 Health Sciences, Faculty of Sport and Health Sciences, University of Jyvaskyla, Jyväskylä, Finland

## Abstract

In normal ageing, structural and functional changes in the brain lead to an altered processing of sensory stimuli and to changes in cognitive functions. The link between changes in sensory processing and cognition is not well understood, but physical fitness is suggested to be beneficial for both. We recorded event-related potentials to somatosensory and auditory stimuli in a passive change detection paradigm from 81 older and 38 young women and investigated their associations with cognitive performance. In older adults also associations to physical fitness were studied. The somatosensory mismatch response was attenuated in older adults and it associated with executive functions. Somatosensory P3a did not show group differences, but in older adults, it associated with physical fitness. Auditory N1 and P2 responses to repetitive stimuli were larger in amplitude in older than in young adults. There were no group differences in the auditory mismatch negativity, but it associated with working memory capacity in young but not in older adults. Our results indicate that in ageing, changes in stimulus encoding and deviance detection are observable in electrophysiological responses to task-irrelevant somatosensory and auditory stimuli, and the higher somatosensory response amplitudes are associated with better executive functions and physical fitness.

## Introduction

Normal ageing is accompanied by a degeneration of brain structure^1^ and changes in sensory processing, memory, and executive functions^2,3^. Age-related atrophy of brain tissue, together with changes in neural transmission, result in a reorganisation of neural circuits and compensatory brain activity, which eventually leads to alterations in cognitive performance^3,4^. Since the changes in the nervous system precede those in behaviour, event-related potentials (ERPs) that reflect the brain’s sensory-cognitive functions are promising tools to detect early ageing-related cognitive deterioration^5^.

Mismatch negativity (MMN), which is an automatic ERP response to stimulus changes, indexes cognitive decline in normal ageing as well as in different neuropsychiatric, neurological, and neurodevelopmental disorders^6,7^. The MMN is elicited in the oddball condition, where rare deviant stimuli are interspersed with repetitive standard stimuli^8^. The change detection the MMN reflects is based on the comparison process between the memory trace formed by the standard stimuli and deviant stimulus input^9^. The MMN occurs usually 150–250 ms post-stimulus^8^. The MMN was first discovered in the auditory sensory modality^10^, and changes in stimulus intensity, frequency, or location are reflected by the MMN amplitude^9^. The MMN has also been demonstrated to respond to changes in somatosensory^11–14^, visual^15,16^, and olfactory^17^ stimuli.

In the auditory modality, changes in stimulus duration and frequency have primarily been used to study age-related alterations in sensory processing^18^. The auditory MMN (aMMN) amplitude to changes in frequency^19–21^ and duration^21,22^ is attenuated in older adults compared to young adults. The amplitude of aMMN related to changes in stimulus duration and inter-stimulus intervals may be associated with impaired cognitive performance, especially in verbal memory and executive functions^23–25^.

Somatosensory change detection paradigms and their associations with ageing are less studied than their auditory counterparts. Only one study has applied the somatosensory mismatch response (sMMR) to investigate pre-attentive change detection in older adults. In the study, it was found that the sMMR to electrical pulses applied to different fingers was altered in a group of healthy older adults compared to young adults^26^. The sMMR was evident in young adults in early and late latency ranges (180–220 ms and 250–290 ms after stimulus onset, respectively), while the early sMMR was absent and the late sMMR was attenuated in older adults.

In addition to MMN, other ERP components elicited in the passive oddball condition—N1, P2, and P3a—are shown to be sensitive to ageing^27–29^. Auditory N1 reflects automatic stimulus encoding and is elicited in the auditory cortex approximately 100 ms after tone onset^30^. A recent study reported increased N1 responses to repetitive standard stimuli in older compared to young adults, reflecting an age-related decrease in sensory inhibition^31^. P2, which is mostly studied in the auditory modality and typically peaks at around 150–250 ms post-stimulus, is involved in stimulus classification and the processing of task-irrelevant stimuli^32,33^. The effects of ageing on P2 are inconclusive. Notably, the only study reporting ageing-related decrease of P2 amplitude to frequency changes^34^ used a passive oddball condition, where stimuli are outside of the attention of the participant. The studies reporting the opposite effects^35,36^ or no effects related to ageing^37,38^ used active oddball tasks, where the stimuli are attended to. In a passive oddball condition, P3a peaks at approximately 250–500 ms and usually has a fronto-central scalp topography. This reflects the automatic re-orienting of attention that follows the pre-attentive change detection and may also include conscious recognition of the stimuli^28^. In normal ageing, auditory P3a amplitude typically decreases^22,39,40^, and its latency increases^41^.

Here, we compared the brain responses of 38 young adults and 81 older adults to study the effects of ageing on sensory-cognitive functions in a passive change detection paradigm in the auditory and somatosensory modality. All participants performed a cognitive assessment, and the older adults also participated in a physical fitness measurement. Previous literature suggests that higher physical activity is linked to better cognitive performance^42^ and to better cortical sensory processing reflected by ERPs^43–45^ in older adults. Thus far, no ageing study has combined ERPs, cognition, and objective measures of physical fitness, making the current study the first in the field.

We hypothesised that sMMR^26^ and aMMN^18^ are diminished in amplitude in older participants compared to those in young participants. A similar attenuation of amplitude could be found for ERP components following aMMN/sMMR, namely, P2 and P3a^22,34^. We also hypothesised that ERPs correlate with cognitive test scores^23,24^. In older adults, we expected better physical fitness, especially aerobic fitness^46^, to be associated with better cognitive performance and less attenuated ERP amplitudes, since physical activity and fitness may mitigate ageing-related cognitive decline^46,47^.

## Results

### Early somatosensory ERP components

**Figure 1:**
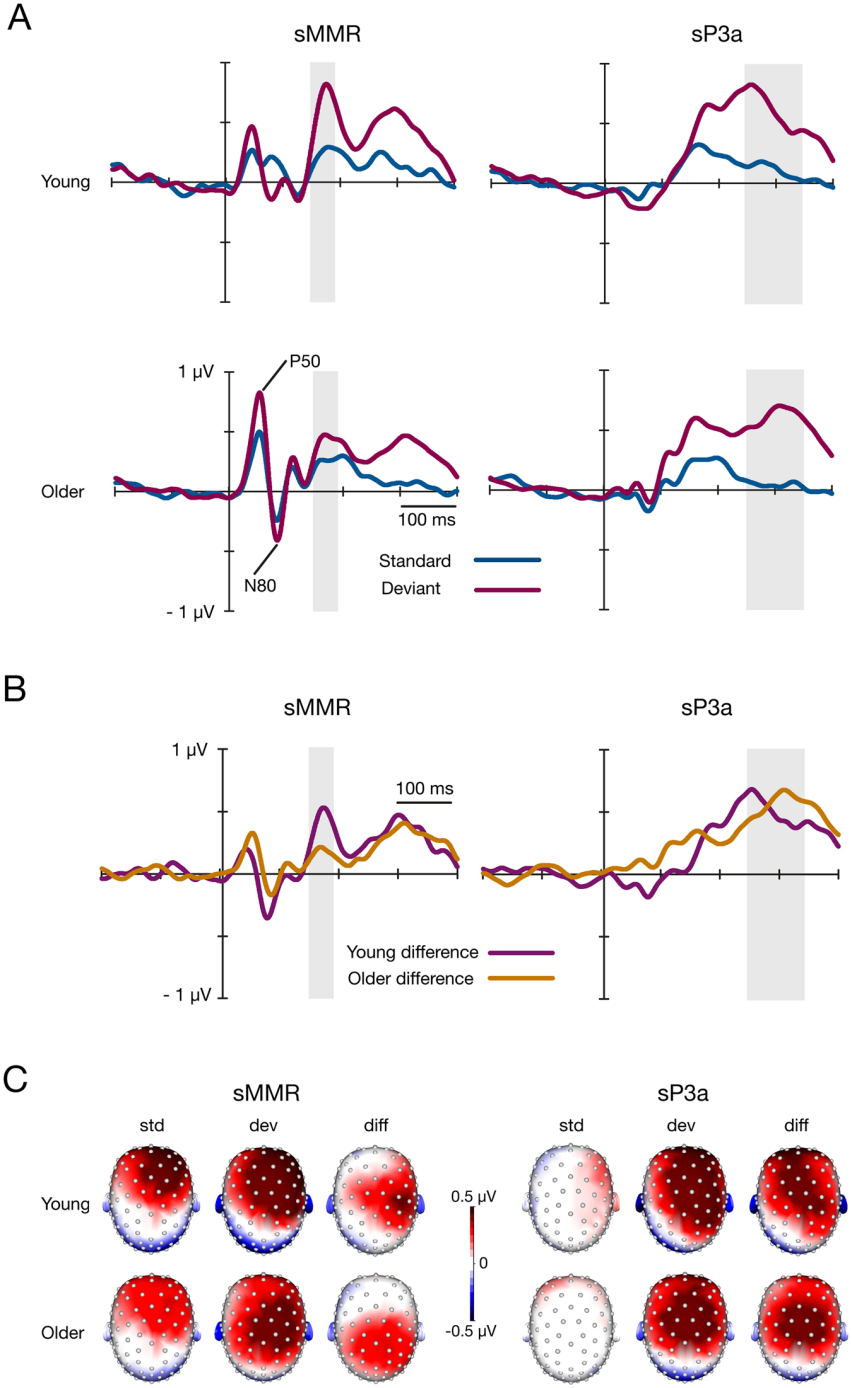
(**A**) Grand-averaged ERPs to somatosensory standard and deviant stimuli for young and older adults and (**B**) the differential waveforms (standard minus deviant) for young and older adults. Waveforms represent averages of the electrode pools applied in the analyses. The grey area shows the latency range of 153–193 ms for sMMR and of 258–358 for sP3a, from where the averaged amplitude values were extracted to analyse each ERP component. (**C**) The scalp voltage distributions of responses to standard (std) and deviant (dev) stimuli and differential responses (diff) (deviants minus standards). The topographic maps are shown as average voltages from 153–193 ms for sMMR and from 258–358 for sP3a. Note, due to keeping the scaling equal throughout, the lateralisation of differential response in older adults is no longer observable in the scalp topography of sMMR.

P50 and N80 peak amplitudes were analysed due to apparent differences in grand-average waveforms between the age groups (Fig. 1). The mean amplitude of P50 and N80 were larger in older participants than in young participants for both standard and deviant stimuli (Table 1, Fig. 1a). Within both age groups, the amplitudes of P50 and N80 were larger for deviants than for standards. The age differences on P50 and N80 were not significant after controlling for the stimulus intensities, indicating that the group differences are due to higher stimulus intensities in older adults than in young adults (Table 1). The latency of the P50 deviant stimuli response was prolonged in older participants compared to young participants, as follows: mean for young adults, 47 ± 9 ms; mean for older adults, 51 ± 7 ms; mean difference between the groups, 4.0 ms; standard error of mean (SEM), 1.7; F = 6.78, df = 1, df error = 117; *p* = 0.010, partial eta squared (*η*_*p*_^2^) = 0.055. This result remained significant after controlling for stimulus intensities, as follows: F = 4.22, df = 1, df error = 115; *p* = 0.042, *η*_*p*_^2^ = 0.035. No other effects on latency were found.

**Table 1:** Results of ANCOVA of early somatosensory ERP components in response to deviant and standard stimuli in younger and older adult groups.

	Mean amplitude (µV) ± SD		Mean difference	Age group main effect			Age group effect with stimulus intensities as covariates		
	Young	Older	Mean [SEM]	*F* (*df*, *error df*)	*p*	*η*_*p*_^2^	*F* (*df*, *error df*)	*p*	*η*_*p*_^2^
**P50**									
**std**	0.58 ± 0.45	0.83 ± 0.60	0.23 [0.10]	4.57 (1,117)	0.035*	0.038	0.03 (1,115)	0.863	<0.001
**dev**	0.82 ± 0.51	1.24 ± 0. 75	0.42 [0.12]	9.55 (1,117)	0.002**	0.075	0.30 (1,115)	0.592	0.003
**N80**									
**std**	0.06 ± 0.41	0.42 ± 0.48	0.37 [0.08]	16.77 (1,117)	<0.001***	0.125	3.26 (1,115)	0.074	0.028
**dev**	0.30 ± 0.70	0.62 ± 0.61	0.32 [0.13]	6.56 (1,117)	0.012*	0.053	0.88 (1,115)	0.350	0.008

Stimulus intensities for little finger and forefinger were used as covariates. SEM, standard error of mean; SD, standard deviation; df, degrees of freedom; *η*_*p*_^2^, partial eta squared; *p*, statistical significance; *p < 0.05; **p < 0.01; ***p < 0.001.

### Later somatosensory and auditory ERP components

Topographic maps for somatosensory responses (Fig. 1) show a positive polarity sMMR^26^ and sP3a^28^ similar to those reported earlier in the somatosensory modality^12,13,26^. sMMR topography illustrated contralaterally localised positivity for standard and deviant stimuli in both age groups although lower amplitude in the older group. In the group of young adults, both sMMR and sP3a to deviant stimuli elicited activity at fronto-central electrode sites, while in the older adults the activation was prominent only in central electrode sites.

**Figure 2:**
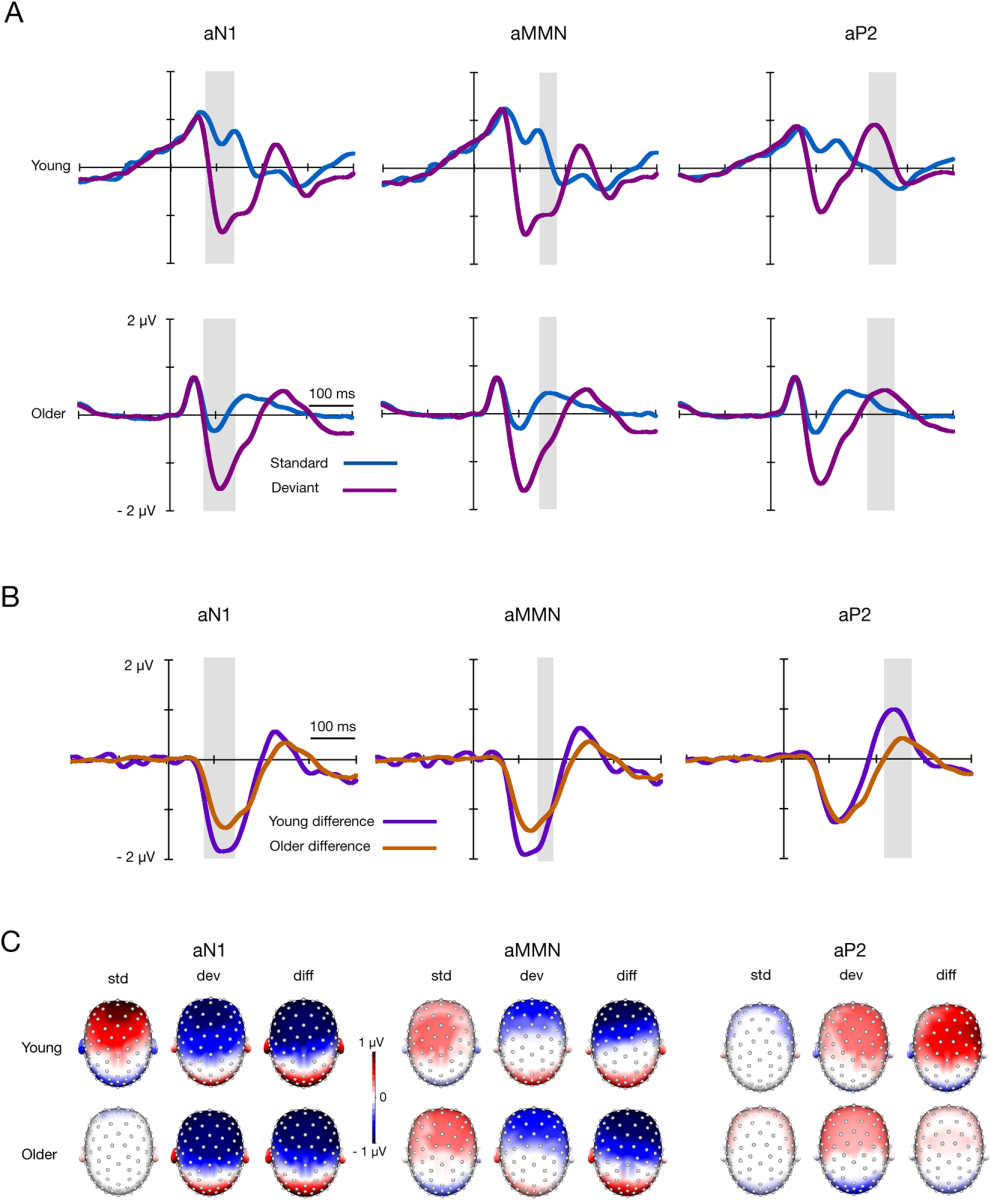
(**A**) Grand-averaged ERPs to auditory standard and deviant stimuli for young and older adults and (**B**) the differential waveforms (standard minus deviant) for young and older adults. Waveforms represent averages of the electrode pools applied in the analyses. The grey area shows the latency range of 88–138 ms for aN1, of 139–189 ms for aMMN, and of 208–280 ms for aP2, from where the averaged amplitude values were extracted to analyse each ERP component. (**C**) The scalp voltage distributions of responses to standard (std) and deviant (dev) stimuli and differential responses (diff) (deviant minus standard). The topography maps are shown as average voltages from 88–138 ms for aN1, 139–189 ms for aMMN, and 208–280 ms for aP2.

Topographic maps for the auditory responses show typical aN1^31^, aMMN^8^, and aP2^33^ responses with most of the activity in the frontal electrode sites (Fig. 2). There were no clearly observable differences in auditory grand-averaged topographies between the groups other than those caused by an amplitude difference in the standard response (Fig. 2).

A two-way repeated measures multivariate analysis of variance (MANOVA) revealed the main effect of age group for amplitude values in the sMMR time window (mean for young adults, 0.48 µV; mean for older adults, 0.36 µV; amplitude values averaged for the standard and deviant stimuli) and aN1 time window (mean for young adults, 0.25 µV; mean for older adults −0.76 µV; amplitude values averaged for the standard and deviant stimuli) (Table 2, Figs 1 and 2). For all components—sMMR, sP3a, aN1, aMMN, and aP2ta—the main effect of stimulus type was found, indicating that the amplitudes to the deviant stimuli were larger than those to the standard stimuli for all components (Table 3, Figs 1 and 2). An interaction effect of stimulus type × age group was found for sMMR, aN1, and aP2 (Table 2, Figs 1 and 2). The following independent samples *t*-tests (two-tailed, bootstrap statistics) showed that the deviant responses in the sMMR analysis window were attenuated and that the standard responses for aN1 and aP2 were enlarged in older adults compared to young adults (Table 3). The interaction effect of stimulus type × age group for sMMR remained significant after controlling for stimulus intensity (*p* = 0.019); similarly, the interaction effect for aP2 was significant when controlling for hearing threshold (*p* = 0.005), but hearing threshold as a covariate decreased the *p* value of the interaction effect of stimulus type × age group for aN1 (*p* = 0.055).

**Table 2:** Results of the two-way repeated measures MANOVA of later somatosensory and auditory ERP components in response to deviant and standard stimuli in young and older adult groups.

	Age group Main effect			Stimulus type Main effect			Stimulus type × Age group Interaction		
	*F* (*df*, *error df*)	*p*	*η*_*p*_^2^	*F* (*df*, *error df*)	*p*	*η*_*p*_^2^	*F* (*df*, *error df*)	*p*	*η*_*p*_^2^
sMMR	2.73 (1,117)	<0.001***	0.023	24.57 (1,117)	<0.001***	0.174	5.24 (1,117)	0.024*	0.043
sP3a	0.32 (1,117)	0.575	0.316	83,40 (1,117)	<0.001***	0.416	0.12 (1,117)	0.730	0.001
aN1	16.52 (1,117)	<0.001***	0.124	324.09 (1,117)	<0.001***	0.735	10.63 (1,117)	0.001***	0.083
aMMN	1.93 (1,117)	0.168	0.016	127.35 (1,117)	<0.001***	0.521	0.37 (1,117)	0.541	0.003
aP2	1.72 (1,117)	0.192	0.015	44.20 (1,117)	<0.001***	0.274	11.65 (1,117)	0.001***	0.091

Df, degrees of freedom; *η*_*p*_^2^, partial eta squared; *p*, statistical significance; *p < 0.05; **p < 0.01; ***p < 0.001.

**Table 3:** Mean amplitude values and standard deviantions and results of the independent samples *t-*tests (two-tailed, bootstrapped with 1000 iterations) comparing the response amplitudes between the groups of young and older adults in the later somatosensory and auditory ERP components in response to standard and deviant stimuli.

	Mean amplitude (µV) ± SD		Difference between young adults and older adults				
	Young	Older	Mean [SEM]	*95% CI*	*t* (*df*)	*p*	*d*
sMMR							
std	0.26 ± 0.40	0.26 ± 0.35	<0.01 [0.07]	−0.14 to 0.15	0.01 (117)	0.992	0.02
dev	0.70 ± 0.74	0.42 ± 0.63	0.28 [0.14]	0.01 to 0.56	2.16 (117)	0.043*	0.40
aN1							
std	0.62 ± 0.74	−0.16 ± 0.70	0.78 [0.15]	0.48 to 1.08	5.54 (117)	0.001***	1.02
dev	−0.11 ± 0.72	−1.36 ± 0.85	0.25 [0.15]	−0.06 to 0.53	1.57 (117)	0.105	0.30
aP2							
std	−0.18 ± 0.45	0.18 ± 0.39	0.36 [0.08]	0.19 to 0.52	4.48 (117)	0.001***	0.83
dev	0.60 ± 0.83	0.43 ± 0.56	0.16 [0.15]	−0.12 to 0.47	1.27 (117)	0.298	0.23

SEM, standard error of mean; SD, standard deviation; CI, confidence interval; *d*, Cohen’s *d*; df, degrees of freedom; *p*, statistical significance; *p < 0.05; **p < 0.01; ***p < 0.001.

### Relationships between ERPs, cognitive test scores, and physical fitness measures

Table 4 illustrates the significant correlations within 95% and 99% confidence intervals (CIs). In older adults, the most robust positive correlations (within 99% CI) were found between sMMR and executive functions and between sP3a and walk test performance. These correlations in older adults remain significant (p < 0.05, 99% CI does not include zero) after controlling for age but not for education. Within the young adult group, a robust positive correlation was found between the aMMN and working memory, which remained significant after controlling for age and education (Table 4). In older adults, aMMN correlated neither with any of the cognitive measures nor with physical fitness measures.

**Table 4:** Correlations between cognitive and physical measures and ERPs.

Test	Older adults					Young adults			
	Variable	*r*	*p*	*99% CI*	*95% CI*	*r*	*p*	*99% CI*	*95% CI*
Executive function PC	sMMR (age)	0.299*	0.004	0.001 to 0.594	0.035 to 0.524	*ns*	*ns*	*ns*	*ns*
	sP3a	0.239	0.017	−0.062 to 0.517	0.003 to 0.468	*ns*	*ns*	*ns*	*ns*
	Six-minute walk distance (age, edu)	0.203	0.036	−0.076 to 0.443	0.011 to 0.395	—	—	—	—
Error susceptibility PC	sMMR (edu, age)	−0.276	0.007	−0.491 to 0.021	−0.465 to −0.055	*ns*	*ns*	*ns*	*ns*
Explicit memory PC	aP2 (age, edu)	0.254	0.012	−0.025 to 0.508	0.025 to 0.439	*ns*	*ns*	*ns*	*ns*
Working memory PC	aMMN	*ns*	*ns*	*ns*	*ns*	0.479*	0.001	0.045 to 0.756	0.184 to 0.689
Six-minute walk distance	sP3a (age)	0.319*	0.002	0.062 to 0.548	0.131 to 0.502	—	—	—	—
	Executive function PC (age, edu)	0.284	0.006	−0.050 to 0.531	0.059 to 0.490	—	—	—	—
	sMMR (edu)	0.203	0.036	−0.076 to 0.443	0.011 to 0.395	—	—	—	—
Tapping speed – dominant hand	sP3a	0.272	0.008	−0.009 to 0.533	0.034 to 0.478	*ns*	*ns*	*ns*	*ns*
	sMMR	0.215	0.028	−0.064 to 0.463	0.017 to 0.393	*ns*	*ns*	*ns*	*ns*
	aMMN (age)	−0.229	0.021	−0.477 to 0.033	−0.417 to −0.032	0.417	0.005	−0.026 to 0.715	0.116 to 0.658
Tapping speed – non-dominant hand	sP3a (age)	0.298	0.004	−0.019 to 0.600	0.067 to 0.520	*ns*	*ns*	*ns*	*ns*
	aN1 (age)	−0.252	0.013	−0.481 to 0.009	−0.436 to −0.058	*ns*	*ns*	*ns*	*ns*
	aMMN	*ns*	*ns*	*ns*	*ns*	0.387	0.008	−0.049 to 0.668	0.087 to 0.622

Variables that show correlation at least in one of the groups within 95% CI are listed; those showing significant correlation within 99% CI are marked with *. Age and/or education (edu) in parentheses refers to significant partial correlations after controlling for the mentioned variable. *r*, Pearson’s correlation coefficient (bootstrap statistics with 1000 iterations); *p*, significance (one-tailed); *CI*, confidence interval; *ns*, non–significant; −, not measured within the young adult group.

The walk test performance had a robust negative correlation with total body fat percentage in older adults (two-tailed Pearson’s r = −0.523, n = 79, p < 0.001, 99% CIs = −0.732 to −0.267) and body mass index (BMI) (n = 79, r = −0.462, p < 0.001, 99% CIs = −0.683 to −0.199) and positively correlated with the self-reported weekly physical activity hours (Spearman’s rho = 0.481, n = 74, p < 0.001, 99% CIs = 0.220–0.687).

## Discussion

We measured the ERPs to auditory frequency and somatosensory location changes in an ignore condition in young and older adults. The somatosensory P50, N80, and sMMR and the auditory aN1 and aP2 differed in amplitude between the groups. As expected, within the older group, higher sMMR amplitude showed a robust association with better executive functions, and higher sP3 amplitude was associated with longer walking distance (CI 99%, Table 4). There were also correlations between the auditory brain responses and tapping speed and explicit memory within the older group, but these associations were less robust (CI 95%, Table 4).

Somatosensory MMR was observed as a shift toward positive polarity at 153–193 ms in both age groups, which is in line with prior findings^11–13^. The differential response was larger in the young group than in the older group due to a larger deviant stimulus response amplitude in the young group, as was found in our earlier study^26^, probably indicating attenuated deviance detection in older adults. Since the deviance detection the mismatch response reflects is suggested to be a cortical process^9^, the changes in the sMMR can be expected to be related to the function of the somatosensory cortex. For sP3a, no group differences were found, and the deviant vs. standard differential response was significant in both groups. The pattern of results in the somatosensory modality showing attenuated sMMR, but no changes in amplitude of sP3 suggest that change detection, but not the following automatic shift of attention, is affected in ageing. It is notable, however, that the response latency of sP3a seems to be delayed in the older adults compared to young adults, but the data did not allow a valid statistical analysis to investigate this difference since there were no clear peak for sP3a for each individual, and mean amplitude values were thus applied in the analysis.

In addition to longer latency components, the amplitudes of the early somatosensory P50 and N80 were also larger in the older group than in the young group. This was mainly explained by higher sensory thresholds and thus higher stimulus intensities in older than younger participants. The result cannot be directly compared with the previous results where stimulus conditions (oddball vs. paired-pulse condition) and stimulus properties have been different between the studies^48–50^. Previous studies that have applied paired-pulse stimulus conditions have reported an ageing-related decline in cortical inhibition accompanied with behavioural inhibitory dysfunction^48–50^.

Auditory N1 and P2 were affected by ageing. The aN1 responses to standard stimuli were larger in amplitude in older adults than in young adults, leading to a smaller differential response between standard and deviant stimuli in older than in young adults. This result is similar to that of a recent study in which syllable changes in speech sounds were applied in the non-attentive oddball condition^31^. In our study, aP2 elicited a differential response in both groups, but a larger differential response was observed in young than in older adults, similar to earlier findings with frequency changes^34^. Again, these results indicate a weaker cortical suppression of the response to standard stimuli in older adults compared to young adults.

Unexpectedly, the groups did not differ in the aMMN amplitude although previous studies have demonstrated its attenuation in aged participants^20,21,51^. Sometimes age group differences became non-observable when short ISIs were used^52,53^. Since the MMN reflects change detection based on a comparison process between a transient memory trace formed by standard stimuli and a deviant input, the longer the applied ISI is, the more demanding the comparison process is for the brain^7^. In the current study, the ISI was relatively short, 400–500 ms, which might explain why we did not find group differences in aMMN.

When comparing the ageing-related findings between the two modalities it is notable that the somatosensory change detection, as indexed by the mismatch response, was altered in older adults while there was no such indication in the auditory modality. The somatosensory mismatch response thus seems to be more sensitive in indicating the ageing-related sensory decline than its auditory counterpart. On the other hand, in the auditory modality, ageing-related alterations were observed in response amplitudes of the N1 and P2 components that reflect stimulus encoding. For these components, increased amplitudes in older compared to young adults were found, reflecting that N1 and P2 are indicative of the altered cortical suppression in older adults. There was no evidence on ageing-related changes in the functioning of the attention shift mechanism towards stimulus changes (sP3a) in the somatosensory modality. The auditory stimuli elicited no clear P3a, and therefore ageing-related effects on P3a could not be studied.

Somatosensory, but not auditory, ERP amplitudes correlated robustly (99% CI) with cognitive performance (larger sMMR was associated with better executive functions) and physical fitness (larger sP3a was associated with longer walking distance) in older adults. A less substantial positive correlation was found between executive functions and walking distance. Thus far, no studies have investigated the relationships between ERPs elicited by somatosensory oddball stimuli and both cognition and physical fitness. However, a recent study demonstrated that sMMR is a sensitive indicator of long-term physical activity in young adults^54^. The study compared the brain activity of male twin pairs with discordant physical activity. The more active twin, who also had higher aerobic capacity and lower body fat percentage, produced a lower peak amplitude sMMR. The authors interpreted that active young adults showed better gating of deviant sensory stimuli. In the current study within the older adult group, however, better performance in the walk test was associated with higher sP3a and sMMR amplitude, but there was no correlation with ERPs that more directly reflect sensory gating, namely P50 and N80. Direct comparison of Tarkka *et al*.^54^ with the current data is also hampered by the different methodology to analyse sMMR. Furthermore, P50 and N80 were not analysed in their data and thus the results concerning these components remain open.

Previous aMMN studies with older participants, which employed duration changes as stimuli, reported a correlation between the aMMN amplitude and executive functions and working memory^23,24^. In our data, aMMN to frequency deviations showed no correlations to cognitive tests in older adults. However, within the young adult group, the aMMN amplitude correlated robustly (99% CI) with working memory performance, possibly indicating that a well-functioning auditory sensory memory supports working memory. Since the age groups differed in working memory but not in aMMN, it suggests that decline in working memory functions may precede alterations of the auditory sensory memory in ageing. However, it is possible that the short ISI applied here was not the most optimal in revealing possible ageing-related alterations in the sensory memory. Additionally, aMMN showed some association (95% CI) with psychomotor speed (finger tapping test) in both age groups, alhough the results are inconclusive due to opposite direction correlations between the age groups.

Better performance in the walk test was associated with cognitive functions requiring executive control in older adults. This finding is congruent with the findings of a meta-analysis, which showed that higher physical fitness is associated with better executive functions in older adults^42^. Better performance in the six-minute walk test was associated with a lower body fat percentage, lower BMI, and higher self-reported physical activity levels, indicating that the six-minute walk test was a suitable objective measure of sub-maximal exercise in older adults^55^.

There are some limitations to the present study. A part of the sample of older females was initially recruited for a physical exercise intervention study, which may mean these participants were on average more active than other participants of their age. However, this was balanced by recruiting about the same number of physically passive older females. Obviously, the results of the present study apply to women only. It is also worth noting that the age range in the older group (18 years) is wider than that within the young group (10 years) although most of the results remain stable after controlling the analyses for age (see Table 4). One limitation is that the somatosensory stimulus intensities were adjusted individually, but the auditory stimulus intensities were constant between the participants. Individual adjustment of the somatosensory stimulus intensities is important because it is difficult to find a fixed intensity that is not painful for someone and still discernible for all participants. Since the ERPs were measured to frequency and location changes, not to intensity changes, it might not be critical that the intensities of the auditory stimuli were of individually adjusted. Importantly, most of the results remained the same when controlling the analyses of the somatosensory brain responses for stimulus intensities and of auditory brain responses for hearing thresholds.

Due to the lack of participants’ individual MRI data and suitable head models for the two relatively distant age groups, our data do not allow source analysis to compare the neural generators of the analysed brain responses between the age groups. In the grand average level, the topographies of the electrical fields of the two groups were relatively similar. Future studies should investigate whether the sources of the responses between the age groups are different.

In conclusion, ageing affects the preattentive processing of somatosensory and auditory stimuli. The sMMR indicated attenuated change detection in older adults. The long latency somatosensory brain responses were also associated with executive functions (sMMR) and physical fitness (sP3a). In the auditory modality, brain responses showed an altered encoding of sensory information in older adults, as reflected by larger standard stimulus aN1 and aP2a responses in older than young adults. Together these results suggest that ageing-related cognitive decline is observable both in cortical sensory responses and in behaviour and that physical fitness can help preserve executive functions during ageing.

## Methods

### Participants

Experiments were carried out in spring 2013 and summer 2014 at the University of Jyväskylä. Data were collected from 131 (41 young and 90 older) healthy females. The data of three young and nine older participants were excluded from further analyses due to contaminated electroencephalography (EEG) data or due to a lack of behavioural data or fitness assessment, resulting in the analysis of a total of 38 young and 81 older women. The ages of the young and older participants ranged from 20–30 (mean ± SD, 23.6 ± 2.8) years and 63–81 (68.1 ± 4.4) years, respectively. In terms of educational background, the percentage of young and older adults, respectively, who had completed elementary school only was 1 and 11%; 34 and 46% had completed secondary school only; 26 and 46% had completed lower tertiary school or bachelor’s degrees only; and 37 and 31% had completed master’s degrees or higher academic degree. All participants were right-handed and lacked any history of neurological illnesses or brain operations. The older participants were recruited from the University of the Third Age in Jyväskylä and the Society of the Retired in Jyväskylä as well as through an announcement in the local newspaper. Participants for the 2013 data collection were recruited for a larger study investigating the effectiveness of a 10-week physical exercise intervention. Here, we reported the results of their baseline measurements. For the 2014 data collection, participants who do not exercise regularly or at all were recruited for a single-day measurement. Young adult participants were recruited from the mailing lists of the University of Jyväskylä’s students’ association. Ethical approval for the study was obtained from the ethical committee of the Central Finland Health Care District. Written informed consent was collected from all participants, and all were given either a movie ticket or coffee package as compensation for their efforts. The experiments were undertaken in accordance with the Declaration of Helsinki.

### Cognitive tests

Participants’ cognitive performance was assessed with cognitive tests selected to encompass domains sensitive to cognitive ageing^2^, including executive functions, perceptual speed, and verbal memory (see Supplementary Table S1). Tests were administered by a psychologist or a trained research assistant during a 60-minute session. The characteristics, including cognitive test scores, of the sample are summarised in Table 5.

**Table 5:** Sample characteristics. Difference between the age groups was tested using independent samples *t*-tests (two-tailed, bootstrap statistics).

Characteristics	Young Mean ± SD	Older Mean ± SD	Mean Difference (95% CI)	*p*	*d*
Physical activity and fitness					
Six-minute walk test distance (*metres*, *more* = *better*)	—	580 ± 97			
Percent fat	—	39.3 ± 7.1			
BMI	22.5 ± 2.7	27.1 ± 4.4	4.6 (3.4 to 6.1)	0.001	1.11
Self-reported physical activity (*hrs/week*)	4.1 ± 1.1	3.1 ± 1.4			
Principal components of cognitive test scores (*rotated factor loadings*)					
Executive function	0.89 ± 0.58	0.42 ± 0.87	1.3 (1.0 to 1.6)	0.001	1.55
Error susceptibility	0.07 ± 0.61	0.03 ± 1.14	−0.1 (−0.5 to 0.3)	0.440	0.12
Explicit memory	0.62 ± 0.68	0.29 ± 0.93	0.9 (0.6 to 1.2)	0.001	0.93
Working memory	0.60 ± 0.89	0.28 ± 0.93	0.9 (0.5 to 1.2)	0.001	0.87
Cognitive test scores					
Tapping right (*clicks/10* *s*, *more* = *better*)	53 ± 5	41 ± 5	12 (10 to 14)	0.001	2.28
Tapping left (*clicks/10* *s*, *more* = *better*)	47 ± 5	37 ± 5	10 (8 to 12)	0.001	1.87
TMT-A (*seconds*, *less* = *better*)	25 ± 7	42 ± 14	17 (12 to 22)	0.001	1.29
TMT-B (*seconds*, *less* = *better*)	51 ± 18	96 ± 44	45 (30 to 60)	0.001	1.11
Logical memory (*points*, *more* = *better*)	28 ± 5	22 ± 6	6 (4 to 8)	0.001	0.97
Logical memory delayed (*points*, *more* = *better*)	26 ± 6	18 ± 7	8 (5 to 10)	0.001	1.08
Stroop 1 – reading (*seconds*, *faster* = *better*)	48 ± 7	56 ± 9	7 (4 to 11)	0.001	0.78
Stroop 2 – colour labelling (*seconds*, *less* = *better*)	62 ± 10	78 ± 17	16 (10 to 22)	0.001	1.00
Stroop 3 – inhibition (*seconds*, *less* = *better*)	91 ± 21	138 ± 35	47 (35 to 59)	0.001	1.43
Stroop 2 errors (*points*, *less* = *better*)	1 ± 1	1 ± 2	0.7 (0.1 to 1.3)	0.018	0.40
Stroop 3 errors (*points*, *less* = *better*)	1 ± 1	3 ± 5	1.9 (0.3 to 3.6)	0.012	0.44
Visual reproduction (*points*, *more* = *better*)	37 ± 4	34 ± 5	3 (1 to 5)	0.001	0.61
Visual reproduction delayed (*points*, *more* = *better*)	36 ± 4	30 ± 8	6 (3 to 8)	0.001	0.75
Digit span (*points*, *more* = *better*)	8 ± 2	7 ± 2	1.1 (0.4 to 1.8)	0.002	0.58
Digit span backwards (*points*, *more* = *better*)	7 ± 2	6 ± 2	1.2 (0.5 to 1.8)	0.001	0.66
Digit-letter (*points*, *more* = *better*)	12 ± 3	9 ± 3	2.4 (1.3 to 3.5)	0.001	0.79

SD, standard deviation. *P*, statistical significance; *d*, Cohen’s *d*.

### Assessment of physical fitness

Three measures were used to assess physical fitness among the older adults: BMI, total body fat percentage, and a six-minute walk test^56^. Only BMI was calculated for the young adults. Participants completed all the measures during one day within two weeks of the behavioural tests and EEG experiments. BMI was calculated according to the following formula: ${BMI}=\frac{\mathrm{mass}\,(\mathrm{kg})}{{\mathrm{height}}^{2}(m)}$. Total body fat percentage was measured using dual-energy X-ray absorptiometry (DXA) (Delphi QDR series, Hologic, Bedford, MA, USA) to estimate boneless and muscleless body tissue. Participants were instructed to avoid eating just before the DXA measurement. During the scan, participants lay still on the device for approximately 10 minutes. After the DXA, the participants took part in a six-minute walk test on a 200-metre indoor track, where they were instructed to walk as far as they could for six minutes, and their heart rate was monitored after every minute. The self-reported physical activity was assessed by a five-scale question of weekly hours of medium-intensity (inducing perspiration) activity, as follows: <1, 1–2, 2–3, 3–4, and > 5 hours.

### Stimuli and procedure

During the EEG recording, the participant was seated in a chair in an electrically shielded, dimly lit room and monitored via a video camera. The participants were instructed to avoid all additional body movement, facial expressions, talking, and excessive head movement; to not pay any attention to any stimuli; and to be engaged in the silent movie that was played on a screen at a distance of about 1.5 metres. In both auditory and somatosensory experiments, a run of 1000 stimuli of two types varying in either location (somatosensory) or frequency (auditory) was delivered with a randomly varying stimulus onset asynchrony (SOA) of 400, 450, or 500 ms.The relatively short SOA was selected based on our earlier findings showing ageing-related changes in the amplitude of the sMMR with ISI of 500 ms^26^ providing thus a solid basis for the cross modal investigation. In an oddball condition, ‘standard’ stimuli were frequently presented at a probability of 86%, and rare ‘deviant’ stimuli were presented at a probability of 14%. The somatosensory stimuli were always presented first followed by the auditory stimuli.

Somatosensory stimulation was generated with a constant current stimulator (Digitimer Ltd, model DS7A, Welwyn Garden City, UK). Electrical pulses of 200 µs were delivered via flexible metal ring electrodes moistened with conductive jelly (Technomed Europe Ltd, Maastrich, Netherlands) to the left forefinger and little finger; stimulating the cathode above the proximal phalanx and the anode above the distal phalanx. A piece of gauze was placed on the finger between the electrodes to prevent conductivity between the two electrodes in the same finger. Both fingers, forefinger and little finger, were applied standard and deviant stimuli in all participants with a counterbalanced order across the participants. Stimulus intensities were adjusted independently for each participant, and for both stimulated fingers, by double the intensity of the subjective sensory threshold. The subjective thresholds were determined by stimulating the individual fingers and asking the participants to verbally report when they sensed the stimulation. The stimulation began with very low intensities, continued with higher intensities step by step (in steps of 0.1 mA), and eventually went over the somatosensitivity threshold. The procedure was repeated three times and applied separately for both stimulated fingers. Overall, the stimulus intensities for both forefinger and little finger were greater in the older adults than in the young participants (Table 6), similar to our earlier study^26^ and in line with earlier findings^57^.

**Table 6:** Sensory thresholds and stimulus intensities.

Sensory threshold and intensity	Young mean ± SD	Older mean ± SD	Mean difference (95% CI)	*p*	*d*
Somatosensory					
Forefinger threshold (mA)	15.8 ± 2.8	24.2 ± 6.7	8.4 (6.7 to 10.1)	0.001	1.38
Little finger threshold (mA)	15.5 ± 2.3	22.9 ± 6.1	7.3 (5.8 to 8.8)	0.001	1.33
Forefinger intensity (mA)	31.3 ± 6.0	48.3 ± 13.5	17.0 (13.5 to 20.3)	0.001	1.37
Little finger intensity (mA)	30.4 ± 5.2	45.2 ± 11.7	14.8 (11.6 to 17.6)	0.001	1.38
Auditory					
Hearing threshold right ear 1000 Hz (dB)	3.3 ± 6.2	15.9 ± 12.7	12.9 (9.7 to 16.4)	0.001	1.24
Hearing threshold right ear 500 Hz (dB)	8.2 ± 5.3	21.4 ± 13.2	13.3 (10.3 to 16.8)	0.001	1.14
Hearing threshold left ear 1000 Hz (dB)	5.1 ± 8.3	13.1 ± 11.8	10.0 (6.5 to 14.0)	0.001	0.90
Hearing threshold left ear 500 Hz (dB)	12.9 ± 6.7	23.4 ± 13.4	10.5 (7.3 to 14.6)	0.001	0.90

The differences between age groups were tested with independent samples *t*-tests (two-tailed, bootstrap statistics). SD, standard deviation; CI, confidence interval; *p*, statistical significance; *d*, Cohen’s *d*.

The auditory stimuli were sinusoidal sounds 50 ms in duration with a 10-ms onset and offset time, presented from a loudspeaker placed 90 cm above the participant, at an intensity of 75 dB (sound pressure level [SPL]) and at a frequency of either 1000 Hz or 750 Hz. Both frequencies were applied as standard and deviant stimuli in all participants in a counterbalanced order across the participants. Individual hearing thresholds for 500 and 1000 Hz separately for both ears were tested prior to the experiment with an audiometer (Mediroll SA-51, Mediroll Ltd, Debrecen, Hungary) by starting from very low intensities, going stepwise (5 dB) over the hearing threshold and lowering the intensity again well below the hearing threshold reported by the participant. This procedure was repeated three times and the lowest threshold was recorded.The hearing threshold level was generally higher among the older group than in the young group (Table 6).

### Electroencephalography

The EEG was recorded using a high-impedance amplifier and the 128-channel EGI Sensor Net (Electrical Geodesics Inc., Hydrogel GSN 128, 1.0). Impedances were kept below 80 kΩ throughout the experiment. The sampling rate was 1000 Hz, and data were filtered online from 0.1 to 400 Hz. During the recording, the vertex electrode (Cz) was used as the reference electrode.

### EEG data processing

Brain Vision Analyzer 2.0 software was used to analyse the data (Brain Products Gmph). Eye blinks were removed using the Gratton & Coles method^58^, and channels with excessive noise and insufficient skin contact were interpolated using a spherical spline model. Offline, an average reference was applied. The electrode signals were filtered with a low cut-off of 0.1 Hz and a high cut-off of 20 Hz, both with 24 dB/octave roll-off. In addition, a 50-Hz notch filter was applied. Then, extensively large amplitude values, outside −100 to 100 μV from peak to peak, in the EEG data were rejected, and low activity periods (<0.5 μV of change within a 100-ms range) were removed. The average number of included trials (with responses to deviant and preceding standard stimuli) in the auditory experiment were 134 (min. 83, max. 150) for the older and 134 (min. 110, max. 150) for the young adults and for the somatosensory experiment 132 (min. 83, max. 150) for the older and 134 (min. 106, max. 150) for the young adults. Stimulus-locked time windows of 600 ms, from 200 ms prior to stimulus onset to 400 ms after the stimulus onset, were extracted. A pre-stimulus onset time of 200 ms was determined as a baseline.

Although previous studies have not shown age group differences in the somatosensory oddball condition for the early components (P50, N80)^26^, a visual inspection of the current data indicated potential group differences for P50 and N80. Accordingly, the maximum peak amplitudes at the C4 electrode^13^ and its latency were extracted from time windows of 30–80 ms (P50) and 40–110 ms (N80) after stimulus onset.

To select the regions of interests (time windows and electrode sites) for each of the later ERP components (sMMR, sP3a, aN1, aMMN, and aP2), permutation tests^59^ (4000 permutations) were performed as implemented in BESA Statistics 1.0 software (BESA GmbH) starting with all 128 electrode locations. This process was used to compare the average responses of standard and deviant stimuli in the group of young adults, which was considered a reference groups for the older adult group. The time windows were defined by first finding the time point with the highest *t-*value for each component and then using this time point as the centre of the time window. A 40-ms time window was applied for sMMR, a 50-ms window was applied for aN1 and aMMN, a 72-ms window was applied for aP2, and a 100-ms window was applied for sP3a (see Supplementary Figure S2). The width of the time window was set taking into account the latency of the differential response based on a visual inspection of the grand-averaged waveforms. The applied time windows were 153–193 ms after stimulus onset for sMMR, 258–358 ms for sP3a, 88–138 ms for aN1, 139–189 ms for aMMN, and 208–280 ms for aP2. The applied time windows based on permutation tests fitted well to the latencies of the differential responses as charged by visually observing the grand average waveforms.

The electrodes for the analysis were selected by first finding the electrode with the highest *t*-value in the middle of the each selected time window and then defining the surrounding electrodes (see Supplementary Figure S2). The activity of the electrodes within the region of interest was averaged.

The regions of interests were defined based on the data of the young adults, and the same time windows and electrode locations were used in the analysis for the older participants, since there were no substantial differences between the groups.

### Statistical analysis

To compare differences in the peak amplitude and latency of somatosensory P50 and N80 between the age groups, univariate analysis of variances (ANOVA) was applied.

Due to higher sensory thresholds and thus higher stimulus intensities in the older than in the young adults, univariate analysis of covariates (ANCOVA), was also applied using stimulus intensities to forefinger and little finger as covariates. For the other ERP components, repeated measures MANOVA was used to assess differences in response amplitudes to stimulus types (standard, deviant) between the age groups separately for each response (sMMR, sP3a, aN1, aMMN, and aP2). Stimulus type (standard vs. deviant) was applied in the analysis order to investigate whether possible group differences are associated spesifically to one or both of the stimulus types (see also Fig. 8 in^60^). The mean amplitude values from the component-specific electrode pools were applied in the analysis (see Supplementary Figure S2). For these latter components, response latencies were not analysed because it was not always possible to find clear peaks for each individual and both stimulus types. The same analyses were also run with age and education as covariates.

Whenever a stimulus type × age group interaction was found, differential ERPs (deviant minus standard responses) were calculated separately for the young and older participants, and independent samples *t*-tests (two-tailed, bootstrap statistics with 1000 iterations) were performed to compare the standard and deviant responses between the groups. Effect size estimates are described as partial eta squared (*η*_p_^2^) scores for MANOVA and Cohen’s *d* for *t*-tests.

A principal component analysis (PCA) was applied to reduce the dimensionality of the cognitive test scores within the whole sample. Following an exploratory analysis, an oblimin with Kaiser normalisation rotated PCA resulted in four components (eigenvalue > 1.0), including the scores from 14 cognitive tests (communalities > 0.600, r^2^ = 69.4%), which are listed in Table 1. The principal components (PCs) were labelled executive function, error susceptibility, explicit memory, and working memory (see Supplementary Table S3).

One-tailed Pearson’s correlation coefficients and partial correlations with age and education as covariates were computed within the age groups to examine the relationships between the ERPs (deviant - standard differential response), the PC scores from the cognitive test scores, and physical fitness measures. Bootstrap statistics were performed with 1000 iterations and CIs of 99% and 95%. The threshold for statistical significance was *p* < 0.05.

### Data availability

The datasets generated during and/or analysed during the current study are available from the corresponding author on reasonable request.

## Electronic supplementary material

Supplementary information
