# Supplementary material for: Automatic auditory and somatosensory brain responses in relation to cognitive abilities and physical fitness in older adults
Source: Sci Rep. 2017 Oct 20;7:13699. doi: 10.1038/s41598-017-14139-9 (PMC5651800; doi:10.1038/s41598-017-14139-9)
Supplement: Supplementary file 1 — Supplementary information [file 41598_2017_14139_MOESM1_ESM.pdf]

Supplementary material

Automatic auditory and somatosensory brain responses in relation to cognitive abilities and physical fitness in older adults

Authors: Juho M. Strömmer[1*], Nele Põldver[2], Tomi Waselius[1], Ville Kirjavainen[1], Saara Järveläinen[1], Sanni Björksten[1], Ina M. Tarkka[3], Piia Astikainen[1]

Author's Affiliations: 1. Department of Psychology, University of Jyvaskyla, Jyvaskyla, Finland; 2. Institute of Psychology, Doctoral School of Behavioural, Social and Health Sciences, University of Tartu, Tartu, Estonia; 3. Health Sciences, Faculty of Sport and Health Sciences, University of Jyvaskyla, Jyvaskyla, Finland.

Corresponding Author:

Juho Strömmer, MA

Department of Psychology

University of Jyväskylä

P.O. Box 35, FI-40014

Jyväskylä

Finland

Tel: +358 (0) 44 287 1873

Email: juho.strommer@jyu.fi

Supplementary Table S1. Cognitive tests tests and their characteristics.

Test	Reference
**The Stroop Colour-Word** Test is a frontal lobe task, testing executive functions. It consists of three separate A4-sheets; the first comprehends list of colour words written in black. The participant was asked to read out loud all the words. In the second sheet, there are 'Xs' printed in colour, and the participant was asked to name the ink colours. Lastly, the participant was handed a sheet with colour words printed in incongruent colours. The task was to name the colour the word was written in, prompting inhibition to read out loud the written word. All lists were instructed to be read as fast as possible, avoiding mistakes.	Alvarez, J. A. & Emory, E. (2006). Executive function and the frontal lobes: A meta-analytic review. *Neuropsychol. Rev. 16*, 17–42.
**The logical memory task** (WMS-R) was used to assess immediate and delayed auditory memory, and declarative memory. Participant was told a short story and the participant was asked to repeat it immediately as accurately as possible. Hereupon the recall another story was told, which was followed by its immediate recall. The participant was informed that they would be asked to recall the stories later on. In approximately one hour, in-between EEG-blocks, the participant was asked to repeat the story anew.	Elwood, R. W. (1991). The Wechsler Memory Scale-Revised: Psychometric characteristics and clinical application. *Neuropsychol. Rev. 2*, 179–201.
**Visual reproduction task** (WMS-R) assesses memory for nonverbal visual stimuli. It includes series of five images, where each is shown for ten seconds. After presentation, the participant was asked to draw the image from memory. In the delayed task, the participant was asked to reproduce the images in no particular order.	Elwood, R. W. (1991). The Wechsler Memory Scale-Revised: Psychometric characteristics and clinical application. *Neuropsychol. Rev. 2*, 179–201.
In the **digit span task**, the participant was told a random sequence of numbers, which were asked to repeat. If the recall was correct, the sequences eventually grew in length. Backward digit span task required the participant to repeat the told sequence in backward order, involving processing of the digits in the working memory.	Ramsay, M. C. & Reynolds, C. R. Separate digits tests: a brief history, a literature review, and a re-examination of the factor structure of the Test of Memory and Learning (TOMAL). *Neuropsychol. Rev. 5*, 151–71 (1995).
In **letter-number sequencing task** the participant was told sequences which included letters and numbers. The participant was asked to repeat the characters, first numbers in numerical order, from the smallest to the highest, and then letters in alphabetical order.	Crowe, S. F. (2000). Does the letter number sequencing task measure anything more than digit span? *Assessment 7*, 113–7.
**Trail Making Test A** (TMT-A) assesses basic attention. Participant was asked to connect 25 numbers in ascending order on an A4-paper without lifting the pencil. **Trail Making Test B** (TMT-B) requires divided attention. The paper included both numbers and letters. The participant was asked to connect numbers and letters by turns in ascending and alphabetical order. Both tasks were asked to complete as fast as possible, yet avoiding mistakes. TMTs assess attention and psychomotor speed. TMT-B is cognitively more demanding and it requires good executive functioning due to the simultaneous processing of two concepts.	Bowie, C. R. & Harvey, P. D. (2006). Administration and interpretation of the Trail Making Test. *Nat. Protoc. 1,* 2277–2281.
**The finger tapping task** was conducted in order to assess participant's psychomotor speed and motor control. In the task, the participant pressed a button on a mechanical tally counter with their thumb. The aim was to tap as many times as possible in 10 second span. The task was completed with the left hand three times consecutively, and then with the right hand. The scores for each hand were averaged across the three trials.	Ruff, R. M. & Parker, S. B. (1993). Gender- and age-specific changes in motor speed and eye-hand coordination in adults: normative values for the Finger Tapping and Grooved Pegboard Tests. *Percept. Mot. Skills 76*, 1219–1230.

Supplementary Figure S2. HydroCel Geodesic Sensor Net 128-channel electrode cap (EGI, OR, USA).

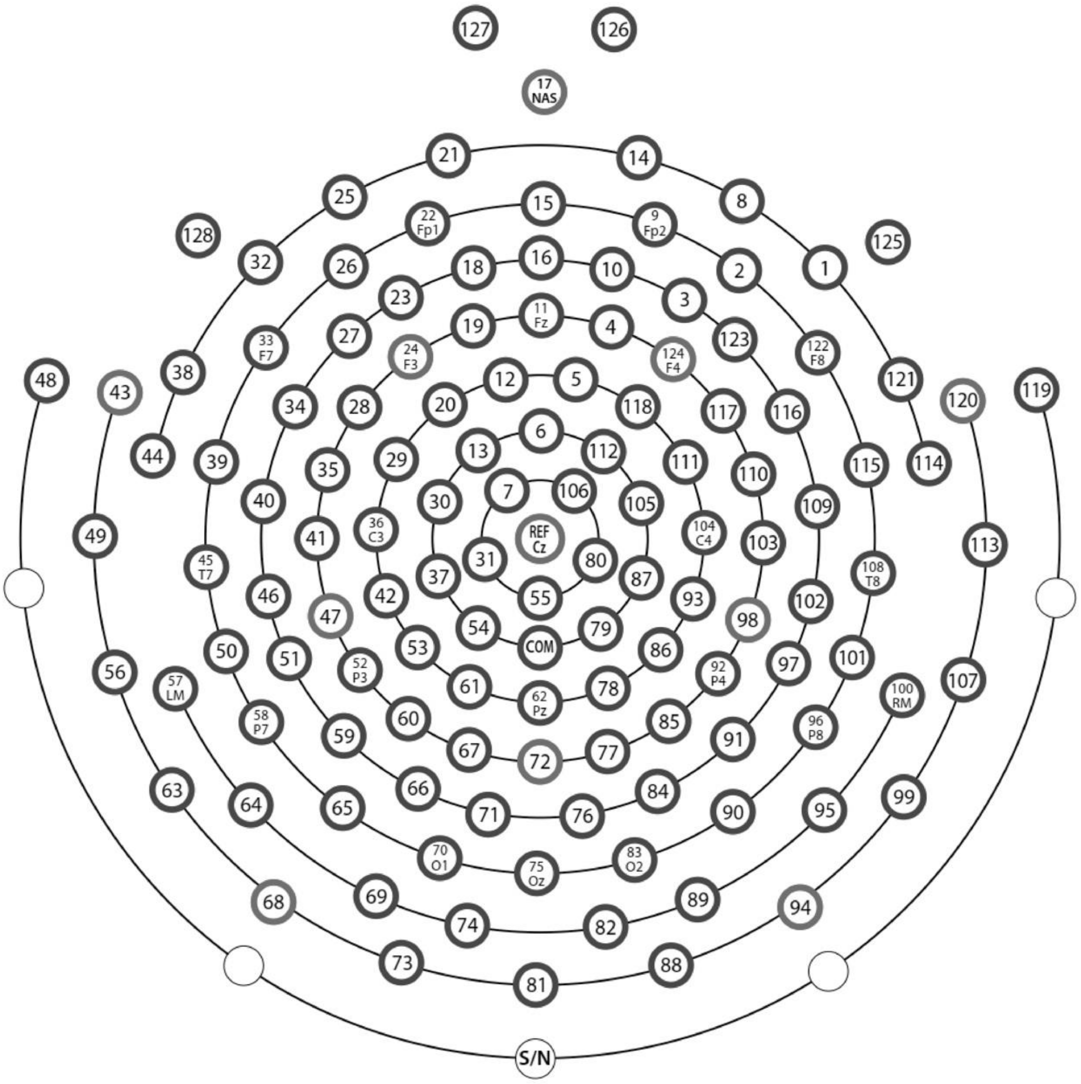

ERP component	Latency range (ms)	Electrode pool (EGI HydroCel)
sMMR	153–193	87, 104, 105, 110, 111
sP3a	258–358	7, 31, 80, 106
aN1	88–138	11, 12, 18, 19, 24
aMMN	139–189	10, 11, 16, 18
aP2	208–280	6, 105, 106, 111, 112

Supplementary Table S3. Principal component loadings rotated with oblimin with Kaiser Normalization (converged in 16 iterations) on cognitive test scores.

	Principal component			
	Executive function	Error susceptibility	Explicit memory	Working memory
Stroop1	-.83	-.209	.102	.132
TMTA	-.769	.134	-.107	.075
Stroop2	-.668	.197	.116	-.264
Stroop3	-.569	.271	-.174	-.27
TMTB	-.438	.051	-.232	-.361
Errors Stroop 2	-.015	.742	-.109	-.167
Errors Stroop 3	-.122	.664	-.209	-.033
Visual reproduction delayed	-.013	-.158	.894	.006
Visual reproduction	-.021	-.161	.874	-.118
Logical memory delayed	.157	.442	.594	.307
Logical memory	.12	.529	.53	.306
Digit span points	-.018	-.073	-.238	.923
Digit-letter	.151	-.031	.056	.745
Digit span backwards	-.106	.033	.256	.703
